# Medical Students and COVID-19: Knowledge, Attitudes, and Precautionary Measures. A Descriptive Study From Jordan

**DOI:** 10.3389/fpubh.2020.00253

**Published:** 2020-05-29

**Authors:** Ashraf I. Khasawneh, Anas Abu Humeidan, Jomana W. Alsulaiman, Sarah Bloukh, Mohannad Ramadan, Tariq N. Al-Shatanawi, Hasan H. Awad, Waleed Y. Hijazi, Kinda R. Al-Kammash, Nail Obeidat, Tareq Saleh, Khalid A. Kheirallah

**Affiliations:** ^1^The Department of Basic Medical Sciences, Faculty of Medicine, The Hashemite University, Zarqa, Jordan; ^2^Department of Pathology, Microbiology and Forensic Medicine, School of Medicine, The University of Jordan, Amman, Jordan; ^3^Department of Pediatrics, Faculty of Medicine, Yarmouk University, Irbid, Jordan; ^4^School of Medicine, Jordan University of Sciences and Technology, Irbid, Jordan; ^5^Department of Public Health, Faculty of Medicine, Al-Balqa Applied University, Al-Salt, Jordan

**Keywords:** COVID-19, knowledge, attitude, precautionary measures, stigma, medical students, Jordan

## Abstract

The recent coronavirus disease (COVID-19) pandemic is associated with increasing morbidity and mortality and has impacted the lives of the global populations. Human behavior and knowledge assessment during the crisis are critical in the overall efforts to contain the outbreak. To assess knowledge, attitude, perceptions, and precautionary measures toward COVID-19 among a sample of medical students in Jordan. This is a cross-sectional descriptive study conducted between the 16th and 19th of March 2020. Participants were students enrolled in different levels of study at the six medical schools in Jordan. An online questionnaire which was posted on online platforms was used. The questionnaire consisted of four main sections: socio-demographics, sources of information, knowledge attitudes, and precautionary measures regarding COVID-19. Medical students used mostly social media (83.4%) and online search engines (84.8%) as their preferred source of information on COVID-19 and relied less on medical search engines (64.1%). Most students believed that hand shaking (93.7%), kissing (94.7%), exposure to contaminated surfaces (97.4%), and droplet inhalation (91.0%) are the primary mode of transmission but were indecisive regarding airborne transmission with only 41.8% in support. Participants also reported that elderly with chronic illnesses are the most susceptible group for the coronavirus infection (95.0%). As a response to the COVID-19 pandemic more than 80.0% of study participants adopted social isolation strategies, regular hand washing, and enhanced personal hygiene measures as their first line of defense against the virus. In conclusion, Jordanian medical students showed expected level of knowledge about the COVID-19 virus and implemented proper strategies to prevent its spread.

## Introduction

Coronaviruses (CoVs) belong to the *Nidovirales* order of the *Coronaviridae* family that are positive-sense single stranded non-segmented RNA viruses. CoVs are divided based on their antigenicity into four groups: alpha-, beta-, gamma-, and delta CoVs (1, 2). All four groups infect primarily mammals and birds and are associated with deadly illnesses that greatly impacted poultry industry (3). Alpha- and beta-CoVs infect humans as well and cause a wide variety of infections ranging from common cold seen with 229E and OC43 CoVs, to croup, bronchiolitis, and pneumonia seen with NL63 and HKU1 (4, 5). Some CoVs, which were considered enzootic infections, have jumped across animal-human species barrier to become a zoonotic infection affecting humans. CoVs, such as the Severe Acute Respiratory Syndrome (SARS) and the Middle East Respiratory Syndrome (MERS), caused by SARS-CoV and MERS-CoV, consecutively, led to virulent infections in humans (6, 7). The SARS outbreak occurred in Southern China in November 2002 and spread to 17 countries infecting 8,089 people with a case-fatality rate of 9.6% (8). MERS, which occurred in 2012 in Saudi Arabia and spread to 21 countries around the globe, infected 2,506 people with 34.0% case-fatality rate (9). Despite having these near pandemic infections no specific antiviral drug or vaccine has been made available for coronaviruses.

In 2020, a new global pandemic has emerged, caused by a new strain of CoV called SARS-CoV-2. This pandemic started in Wuhan, China in December 2019, possibly due to cross-species transmission (10), and involved almost every country in the world causing mostly mild upper respiratory tract symptoms and in a minority of cases lower respiratory tract infections (LRTI) called coronavirus disease-19 (COVID-19) (11, 12). As of May 25th, 2020, more than 5,305,000 cases were reported and more than 342,000 deaths with a case fatality rate of 6.4% (13). The SARS-CoV-2 virus is different from its previous predecessors in that it is highly contagious and easily transmitted from human to human via respiratory droplets and direct contact which led to this enormous number of infected people (14). The day-to-day numbers are still on the rise especially in Europe, and the magnitude of rising numbers of new cases and deaths is hitting the global population hard.

Multiple studies have emerged assessing the virologic characteristics and clinical consequences of COVID-19 (15, 16); however, not enough studies focused on exploring the knowledge, perceived severity and controllability of the COVID-19 among the communities living this pandemic. The knowledge and behavior assessment of the public toward such outbreaks is essential, especially due to the large amount of misconceptions and false information that are circulating on social media in regard to transmission of the disease and methods of acquisition (17). This is of importance to healthcare professionals, service providers and medical sciences students. Such assessments have proven useful as an important means in the education and raising awareness of best practice in previous viral outbreaks including SARS, MERS, and Ebola (18–20).

Jordan, similar to other countries in the world, is suffering from an increasing number of COVID-19, which have invited the government to enforce martial law leading to a national curfew on March 21st, 2020. As of May 25th, the number of confirmed SARS-CoV-2 infections was 711 cases and 9 related deaths (www.corona.moh.gov.jo). Jordan has six medical schools spread across the country with more than 10,000 enrolled students currently under training. Despite major closure of public universities, several medical students are actively volunteering in their communities and local hospital to provide medical assistance and guidance to the public. Accordingly, measuring the levels of knowledge and attitude in the medical student subpopulation is invited. This study represents the first evaluation of COVID-19 outbreak's knowledge, attitudes, and precautionary measures amongst medical students in Jordan.

## Methodology

### Sample and Data Collection

A descriptive cross-sectional study design was used in this work. Our sample consisted of medical students from all the six medical schools in Jordan. Students are enrolled in a 6-years program leading to either a Bachelor of Medicine, Bachelor of Surgery (MBBS) or a Medical Doctor (MD) degree. The study utilized an online questionnaire delivered to participants in the period between March 16th and 19th early during the complete shutdown of universities as part of the Jordanian government's effort to control the outbreak, and 2 days prior to the nationwide curfew. The online questionnaire was created on Google Forms and posted on several online platforms at each medical school accessible by medical students at all levels. These platforms are official channels of communication between schools and students. In addition, class representatives for each academic year were involved in distributing the questionnaire link to students directly. The total population of the study was estimated to be around 10,000 medical students among the six universities. The total number of participants in this study was 1,404 medical students (Table 1). The percentages of participants by medical school provided in Table 1 reflect the size of medical students from within each medical school in Jordan. This reflects a sample of medical students that is proportionate to the size of medical students within each school in Jordan.

**Table 1:** Sociodemographics of study participants.

	**Number**	**Percent**
**Gender**			
Female	836	59.5
Male	568	40.5
Total	1,404	100.0
**University**			
Al-Balqa Applied	117	8.4
Hashemite	192	13.7
Jordan	550	39.1
JUST	362	25.8
Mutah	62	4.4
Yarmouk	121	8.6
Total	1,404	100.0
**School year**			
1st year	145	10.3
2nd year	348	24.8
3rd year	343	24.5
4th year	264	18.8
5th year	176	12.5
6th year	128	9.1
Total	1,404	100.0

### Tools

The online questionnaire consisted of four main divisions: socio-demographics, sources of information, knowledge, and attitudes regarding COVID-19, and precautionary measures. Socio-demographics included questions about gender, academic level/year (1st−6th), and university. Sources of information included identifying the main sources of knowledge related to COVID-19. These included social media, internet search engines, medical search engines, official sites, TV news channels, family and friends, healthcare workers, Non-Governmental Organizations (NGOs), and religious leaders. The frequency of use of the above resources was judged based on the participants response to one of the following options: most or times, rarely or sometimes, and never. Participants were then asked to assess their knowledge about potential sources and modes of transmission. The potential sources of transmission asked about included transmission through air, large droplets inhalation, animals, contaminated food, touching contaminated surfaces, skin contact, fecal-oral route, kissing, hand shaking, mother to fetus, blood transfusion, and breast milk. Participants responded to the above questions with likely, I do not know, and unlikely. Questions about the most susceptible group included questions about children, pregnant women, or people with chronic illnesses. Furthermore, we wanted to know the perception of the medical students toward the virus: is it more likely to cause pneumonia than other common cold viruses? Is it likely to transmit the infection to four more people? And is COVID-19 associated with full recovery in 90.0% of the cases? We also questioned the role of masks in protecting healthy and infected people against COVID-19, the role of a vaccine in preventing the spread of COVID-19 virus, and whether a COVID-19 infected person and their family should be avoided? Stigma related questions included in this study asked whether participants would disclose themselves or their family members if they became infected. We also asked if they would be stressed and have a feeling of insecurity from the hospital setting during the course of treatment, and whether they would hide their illness to avoid isolation. All these questions were answered using a single option of the following: agree, I do not know, and disagree. After that, students were asked about the precautionary measures that they will adopt to prevent themselves and others from getting infected. Precautionary measures included wearing a face mask, washing hands regularly, using disinfectants, paying more attention to personal hygiene, staying at home and avoiding gatherings, paying attention to a balanced diet, disinfecting my phone, avoiding using public transportation and eating outside, avoiding shaking hands and kissing others when greeting them, getting sufficient sleep and fluid intake, monitoring personal physical health, and persuading people to follow the precautionary measures. Participants' responses included: often/always, rarely/sometimes, and never. Finally, participants were asked to express their level of reaction toward the COVID-19 pandemic from the following: I do not care, concerned but not cautious, concerned and cautious, changed many daily preventive behaviors, and became obsessed by the preventive measures.

The survey was pilot tested (*n* = 5 students) and proper modifications were completed before posting to participants. Participation in the study was voluntary and personal identifiers were not collected. The study was approved by the Institutional Review Board of the Hashemite University and Al Balqa' Applied University. Data was imported into Excel for management and then SPSS for analysis. Numbers and percentages were presented for all variables. Frequency distributions were also presented. Chi-square distribution was used to assess potential statistical relationships between sociodemographic and knowledge, attitudes, and precautionary measures. Alpha level of 0.05 was used. Only statistically significant relationships were detected.

## Results

### Sources of Information Related to COVID-19 Among Participants

First, we examined the major sources of information that students used to gain knowledge toward the COVID-19 outbreak. Our analysis identified that the majority of medical students relied on online resources to obtain information including the use of social media platforms (Table 2). Thirty-eight percent of students use social media to gain knowledge all or most of the time, 45.6% use social media occasionally, while only 16.6% never rely on social media as a main source of information. Similarly, 35.0% of students used common online search engines such as Google to look for more information regarding COVID-19, 49.8% used it sometimes and only 15.2% reported no engagement in active online research.

**Table 2:** Sources of information related to COVID-19 among participants.

**Source**	**Frequency of use**		
	**Never**	**Rarely or sometimes**	**Most of the time**
Social media	233	640	531
	16.6%	45.6%	37.8%
Google	213	699	492
	15.2%	49.8%	35.0%
Medical search engine	504	521	379
	35.9%	37.1%	27.0%
Official sites	541	431	432
	38.5%	30.7%	30.8%
News	271	632	501
	19.3%	45.0%	35.7%
Family and friends	612	590	202
	43.6%	42.0%	14.4%
Healthcare workers	562	542	300
	40.0%	38.6%	21.4%
NGOs	305	553	546
	21.7%	39.4%	38.9%
Religious leaders	1,167	215	22
	83.1%	15.3%	1.6%

Unexpectedly, only 27.0% of students used medical databases or medical literature search engines for up-to-date information, and more than a third of students (35.9%) never use these options to obtain appropriate knowledge on the outbreak. Moreover, 38.5% of students never rely on official newsletters or updates issued by the Jordan Ministry of Health, the Center for Disease Control, or the similar. On the other hand, our analysis showed that students rarely or sometimes rely on local or international news for updates on the outbreak (45.0%), while, as expected, the majority of students never relied on shared information by friends or family members (43.6%) or from religious leaders (83.1%). Finally, only 21.4% of students obtained information on COVID-19 outbreak directly from health professionals, while the majority (40.0%) never considered that option.

### Knowledge of Potential Sources of Transmission of COVID-19 Among Participants

Next, we wanted to evaluate the level of knowledge among medical students toward possible sources of COVID-19 transmission (Table 3). Around 42.5% of students thought that animals are likely to be possible sources of transmission. Interestingly, while about half of the students thought that the virus can be transmitted through contaminated food products (53.3%), only 38.6% thought that the fecal-oral route is unlikely to be a source of transmission. Importantly, students were split between believing that the infection with COVID-19 is air-borne or not (41.8 and 48.0%, respectively), while 91.0% were sure that the virus is likely to be transmitted through inhalation of infected droplets. Most students seemed in agreement that the virus is likely to be transmitted through direct physical interaction such as hand shaking (93.7%), kissing (94.7%), skin contact (73.8%), or exposure to contaminated surfaces (97.4%). Lastly, most students indicated that they did not know if the virus is transmissible through blood transfusion (47.6%) or vertically through breast milk (62.0%) or through the placenta/birth canal (50.2%).

**Table 3:** Knowledge of potential sources of transmission of COVID-19 among participants.

**Source**	**Likelihood of transmission**		
	**Likely**	**I don't know**	**Unlikely**
From air	587	143	674
	41.8%	10.2%	48.0%
Large droplets	1277	86	41
inhalation	91.0%	6.1%	2.9%
Animals	597	318	489
	42.5%	22.6%	34.8%
Contaminated food	749	292	363
	53.3%	20.8%	25.9%
Touching contaminated	1,368	23	13
surfaces	97.4%	1.6%	0.9%
Skin contact	1,036	151	217
	73.8%	10.8%	15.5%
Fecal-oral route	498	364	542
	35.5%	25.9%	38.6%
Kissing	1,329	53	22
	94.7%	3.8%	1.6%
Hand shaking	1,315	40	49
	93.7%	2.8%	3.5%
Mother to fetus	322	705	377
	22.9%	50.2%	26.9%
Blood transfusion	401	668	335
	28.6%	47.6%	23.9%
Breast milk	182	871	351
	13.0%	62.0%	25.0%

### Knowledge of Potential Risk Factors and Virulence of COVID-19 Among Participants

After that, we evaluated the level of knowledge among medical students regarding possible risk factors for COVID-19 viral infection (Table 4). Most medical student (95.0%) believed that people with chronic illnesses are highly susceptible to COVID-19. Alternatively, they were split regarding risk in pregnant women and children, as 48.3 and 23.6% of the medical students indicated that pregnant women and children, consecutively, are at an increased risk. Moreover, around 76.4% of students believed that an infected patient can transmit the infection for up to four people at each encounter if precautionary measures were not taken, and that COVID-19, unlike common cold and flu viruses, is more likely to cause pneumonia in infected individuals. While a minority of the students (19.3%) believed that masks are protective against COVID-19 infection, 60.6% of them believed that only COVID-19 infected persons should wear a mask to reduce transmission. On the other hand, 67.1% of the students believed that 90.0% of infected patients would recover spontaneously without the need for medical intervention, and 75.0% of students believed that an effective vaccine would halt COVID-19 spread. Finally, the majority of students agreed that if a person got infected with the virus, they should accordingly be avoided (83.3%). However, fewer students believed that this person's family should be isolated (76.9%).

**Table 4:** Knowledge of potential risk factors and virulence of COVID-19 among participants.

	**Level of Agreement**		
**Statement**	**Agree**	**I don't know**	**Disagree**
Children are at a higher risk	331	241	832
for COVID-19	23.6%	17.2%	59.3%
People with chronic	1,334	46	24
diseases are at a higher risk for COVID-19	95.0%	3.3%	1.7%
Pregnant women are at a	678	614	112
higher risk of COVID-19	48.3%	43.7%	8.0%
Unlike common cold viruses	1,048	275	81
and other strains of Corona viruses, COVID-19 could cause pneumonia	74.6%	19.6%	5.8%
COVID-19 has a high	942	318	144
recovery rate where over 90% of cases recover	67.1%	22.6%	10.3%
One sick person can	1,072	222	110
transmit the disease to about four other people	76.4%	15.8%	7.8%
Wearing a regular mask	271	302	831
prevents getting the disease	19.3%	21.5%	59.2%
Only sick people should be	851	139	414
wearing a mask to prevent the spread of the disease	60.6%	9.9%	29.5%
I believe that a vaccine can	1,053	276	75
prevent the spread of COVID-19	75.0%	19.7%	5.3%
If a person gets COVID-19,	1,169	156	79
he/she should be avoided because of it	83.3%	11.1%	5.6%
If a person gets COVID-19,	1,079	213	112
his/ her family should be avoided because of it	76.9%	15.2%	8.0%

### COVID-19 Stigma

Next, we wanted to study the extent of stigma associated with the infection of COVID-19 (Table 5). Interestingly, when asked if they would want the matter to remain private or secret in case a family member contracted the virus, approximately a third of the students believed that it should remain private or secret or were unsure (15.3 and 15.8%, respectively).

**Table 5:** Stigma regarding COVID-19 according to participants.

	**Level of agreement**		
**Statement**	**Agree**	**I don't know**	**Disagree**
If somebody in my family were to get COVID-19, I would want it to remain private or a secret	215	222	967
	15.3%	15.8%	68.9%
If I got infected, I will be extremely stressed of the way the health-workers, people in hospital, hospitalization process will deal with me	431	385	588
	30.7%	27.4%	41.9%
If I got infected, I would do anything to avoid isolation	59	54	1,291
	4.2%	3.8%	92.0%

Surprisingly, only 41.9% of the surveyed students believed that they would not be extremely stressed of the way that health-care providers and people in the hospital would deal with them as well as by the general hospitalization process. As expected, the vast majority of students did not agree that if they got infected, they would avoid isolation by all means (92.0%). However, it is interesting to note that 59 students said that if they got infected, they would do anything to avoid isolation (4.2%).

### Precautionary Measures Adopted by Students to Fight COVID-19

Finally, we investigated the precautionary measures implemented by students to protect themselves from becoming infected with the coronavirus (SARS-CoV-2) (Table 6). Firstly, regular hand washing, paying more attention to personal hygiene, and staying at home were the three most adopted strategies by the students to protect themselves from becoming infected (>80.0%). Furthermore, more than 70.0% of the students have avoided social kissing, attending public gatherings and using public transport for commuting. Also, an equal proportion has followed social distancing procedures and advised people to take precautionary instructions seriously and implement them. Avoiding eating at restaurants, using disinfectants, and avoiding social hand shaking ranked in the third place after previous measures where they were adopted by more than 65.0% of the students. However, we identified a statistically significant relationship between the use of disinfectants and the year (level) of study of students. Students in the last three (clinical) years were more likely to use disinfectants (72.8%) compared to students in the first three (academic) years of study (66.1%) as a protective measure against getting infected.

**Table 6:** Precautionary measures adopted by the participants to fight COVID-19.

**Precautionary measure**	**Frequency**		
	**Never**	**Rarely/sometimes**	**Often/always**
Wearing a face mask	856	412	136
	61.0%	29.3%	9.7%
Wash hands regularly	24	158	1222
	1.7%	11.3%	87.0%
Use disinfectants	140	298	966
	10.0%	21.2%	68.8%
Pay more attention to	26	199	1179
personal hygiene	1.9%	14.2%	84.0%
Avoid contacting with	150	380	874
certain groups of population	10.7%	27.1%	62.3%
Pay attention to balanced	362	609	433
diet	25.8%	43.4%	30.8%
Cleaning/disinfecting my	287	466	651
phone (screen)	20.4%	33.2%	46.4%
Avoid public gatherings	45	292	1067
	3.2%	20.8%	76.0%
Stay at home as much as	28	209	1167
possible	2.0%	14.9%	83.1%
Avoid eating outside	80	345	979
	5.7%	24.6%	69.7%
Avoid shaking hands when	81	364	959
greeting others	5.8%	25.9%	68.3%
Avoid kissing others when	43	240	1121
greeting them	3.1%	17.1%	79.8%
Avoid using public	104	308	992
transportation	7.4%	21.9%	70.7%
Get sufficient sleep	126	513	765
	9.0%	36.5%	54.5%
Closely monitor personal	164	490	750
physical health	11.7%	34.9%	53.4%
Closely monitor the physical	206	479	719
health of the people around you	14.7%	34.1%	51.2%
Persuade people around	65	355	984
you to following the precautionary guidance	4.6%	25.3%	70.1%
Follow social distancing	57	363	984
procedures	4.1%	25.9%	70.1%
Increase fluid intake	176	582	646
	12.5%	41.5%	46.0%

Getting sufficient sleep, personal health monitoring, and cleaning mobile phones and their screens were seen as less important measures and were adopted by >50.0% of the students, although, students in the last three (clinical) years were reported disinfecting their mobile phones (24.0%) more than students in their first three (academic) years of study (18.0%). The relationship between disinfecting mobile phones and academic-clinical year levels was statistically significant (*P* < 0.05).

Surprisingly, only 9.7% of the students thought of wearing a protective mask as an important measure to prevent coronavirus infection. Again here, we identified a statistically significant relationship between the students view on wearing a protective mask and their year (level) of study. For example, the percentage of students that reported never wearing a mask as a precautionary measure against COVID-19 was higher among students in the first three (academic) years (64.3%) in comparison to their counterparts in the last three (clinical) years of study (56.1%).

Lastly, on March 2nd, 2020 the first case of COVID-19 was reported in Jordan. Reaction of the students toward that varied from carelessness (3.1%) to becoming obsessed with preventive measures (6.8%). Most students (45.4%) showed balanced reaction toward this reporting as they showed concern and as a result became more cautious (Table 7). On the other hand, more than 30.0% of the students have changed their daily habits and focused more on implementing the precautionary measures, while that raised concerns only in 13.1% of the students but without putting into effect any preventive measures.

**Table 7:** Level of reaction of participants toward COVID-19.

	**Frequency**
I don't care	44
	3.1%
I have been concerned but not cautious.	184
	13.1%
I am concerned and cautious.	638
	45.4%
I changed many daily preventive behaviors.	442
	31.5%
I became obsessed by preventive measures.	96
	6.8%

## Discussion

The current descriptive study assessed the knowledge and attitudes of medical students in Jordan regarding COVID-19. Participants were found to have good levels of knowledge regarding COVID-19 as well as positive attitudes toward the disease. Good precautionary measures were also detected among participants. Utilization of medical search engines to seek information about COVID-19; however, was not optimal among participants and their reliance on social media sites was noted.

The participation of medical students in providing care to patients, combined with the high transmissibility of diseases that cause pandemics, puts this subpopulation at higher risk for contracting as well as transmitting the disease. During pandemics such as COVID-19, healthcare systems are put under great pressure, and a shortage of healthcare providers (HCP) can drive the participation of less experienced HCP such as medical students. In addition, medical students are commonly referred to for healthcare advice from family and friends, and have demonstrated better knowledge than students of other branches in relation to healthcare issue (21, 22), which, expectedly, is more advanced in higher-level medical students (23). In the current study, around 570 (40.0%) of the correspondents were medical students in the last 3 years of training, doing clinical rotations in a hospital setting, while 841 (60.0%) were within the first 3 years of training, taking on -campus courses at a university setting.

Our assessment of the sources of information used by medical students to learn about COVID-19, revealed an expected massive reliance on online sources, with only 16.6% of participants never using social media as a source of information. This is in accordance with a similar study in Turkish university healthcare students where social media was a major information source for learning about the influenza pandemic (24), but in slight contrast when examining studies on less covered subjects such as the zika virus epidemic where news outlets seemed to be the main source of information (22). This should alert policy makers to the importance of social media in disseminating information to the public especially in cases of pandemics. We also found that official sites such as the CDC website and medical search engines such as PubMed, which should reflect reliable sources of information, were less commonly used than social media and news channels to obtain information. Our data indicate a need for improving visibility of reliable sources of information, even within a subpopulation that should be more familiar than the public with credible medical websites.

In this study, we also found that commonly described routes of disease transmission such as respiratory droplets, close contact, and exposure to contaminated surfaces were identified by more than 90% of students as likely sources of transmission. Yet 41.8% of students seemed to think that the virus is likely to be transmitted from air, this could be due to a confusion between airborne and respiratory droplets modes of transmission, although an important distinction between the two modes of transmission, currently adopted by the WHO, refers to particles >5–10 μm in diameter as respiratory droplets, while airborne transmission occurs in droplet nuclei which are particles <5 μm in diameter. When asked about other less studied, and not frequently discussed routes of transmission, such as breast milk and mother to fetus transmission, most students (62.0, 50.2% respectively) answered with (I do not know), which is unsurprising considering the little mention of such routes in both general and specialized information sources, and the lack of research on the subject.

Outbreaks of novel infectious pathogens with poorly understood outcomes are often associated with tremendous fear amongst the general public (25). Fear and stigmatization may impact the intentions of an infected individual to seek medical assistance in the right timing which might contribute to increased morbidity and mortality. This is true for a spectrum of previous coronavirus outbreaks and other infectious diseases including SARS, MERS, HIV infection and tuberculosis (26–28). In our analysis, medical students seemed less susceptible to stigmatization as they agreed with isolation measures of self or family members and the reveal if infected cases. However, a small percentage of medical students (15.3%) agreed that potential cases of COVID-19 infections in their families should be kept undisclosed. Moreover, a larger extent (30.7%) expressed fear from the diagnostic and therapeutic approaches in local hospital in case they were infected with COVID-19. This potential, despite minimal, stigma in medical students is likely to reflect larger fear and sense of stigmatization among their university peers among other disciplines and possibly in the general public. Unfortunately, this might hinder the current local and healthcare efforts to contain the outbreak and to provide medical help to those in need.

Assessing knowledge of precautionary measures for contracting the disease is the first step in directing future efforts in the educational process, which have been shown to affect future behavior (29). While precautionary measures such as hand washing (87.0%) and staying at home (83.1%) were adopted by participants, only (9.7%) considered wearing a face mask often. This is in stark contrast with a recent study done in a population of Chinese residents where nearly all of the participants (98.0%) admitted to wearing masks when leaving their homes (30). This could be due to differences in regulations enforced by the state, cultural experience in previous pandemics, and the educational level of the two subpopulations. Participants still confirmed the need for wearing a mask by infected individuals (60.6%), such face mask practices are advised by the WHO and CDC. Better education regarding the need for wearing a face mask is essential, especially considering the recent mask shortage that many areas witnessed following news of the COVID-19 pandemic.

There is a paucity of evidence on assessing knowledge and attitude of medical students toward COVID-19. A recent study by Alzoubi et al. investigating knowledge, attitude, and practices toward COVID-19 pandemic among students was conducted in a single institution in Jordan included 323 medical students (55.6%) which is a small sample compared to the number of students included in our study (31). Most students enrolled in the study (86.0%) were in the pre-clinical years whereas 60.0% of our sample represented pre-clinical years students and 40.0% in the clinical years. The questionnaire used was posted to students through social media platforms, similar to our approach, and targeted medical and non-medical students. Interestingly, no significant differences were noted between both groups. Our study did not include non-medical student controls, however, several clues can be inferred from the comparison of knowledge and attitude of medical students in the first 3 years and their counterparts doing clinical rotation, assuming that they will have limited clinical knowledge. Moreover, the questionnaire in our study covered wider aspects of the knowledge, source of transmission, risk factors, precautionary measures, in addition to evaluating stigma and level of reaction of students toward this pandemic which were not included in Alzoubi et al. study. On the other hand, several results were similar. For example, social media was the main source of information, and most students acknowledged the importance of hand washing in preventing virus spread and transmission. Unlike our results, 68.4% of their participants thought that mask wearing is protective compared to only 19.3% in our study. Another report from Iran targeted final years medical students also included a small sample size (240 students), and less detailed questionnaire (32). Their study showed a negative correlation between preventive behavior and risk perception whereas our study showed concordant relation. Lastly, another study targeting healthcare workers at different geographical locations was conducted using an online application was successful in recruiting 134 medical students only. This study failed to show good knowledge of participants to transmission route and symptom onset but showed that a large number of participants relied on social media as a source for their information about the pandemic (33).

## Conclusion and Recommendations

Overall, medical students in Jordan showed expected levels of knowledge and attitude regarding COVID-19 and reported good precautionary measures. Similar to most reports, obtaining medical information, however, tend to depend more on social media rather than scientific sources. Countries where the epidemic is hitting hard should implement strategies to keep their medical students updated about emerging public health and medical emergencies. Students should also be properly guided to proper sources of information during these times. When push comes to shove, students should also be equipped with medical knowledge, proper attitude, and good precautionary measures. Given current global situation, more frequent utilization of social media by medical schools to spread knowledge become a necessity and plans should be placed to implement such dissemination in early stages of medical and public health emergencies.

## Data Availability Statement

The raw data supporting the conclusions of this article will be made available by the authors, without undue reservation.

## Ethics Statement

The studies involving human participants were reviewed and approved by Institutional Review Board at The Hashemite University and AlBalqa' Applied University, Jordan. Written informed consent for participation was not required for this study in accordance with the national legislation and the institutional requirements.

## Author Contributions

AK wrote the manuscript. AH contributed to manuscript writing and assisted in data interpretation. JA, TA-S, HA, WH, and NO assisted in developing the assay tools and data collection. SB conducted data management, organization, and analysis. MR contributed conceptually to research design. TS and KK supervised all research activities, designed the survey tools, performed data management and analysis, and approved the final version of the manuscript. All authors contributed to the article and approved the submitted version.

## Conflict of Interest

The authors declare that the research was conducted in the absence of any commercial or financial relationships that could be construed as a potential conflict of interest.
